# Effectiveness and safety of Jiuwei Zhenxin granules for treating generalized anxiety disorder: A randomized controlled trial

**DOI:** 10.3389/fpsyt.2022.898683

**Published:** 2022-10-04

**Authors:** Xue Wang, Peiran Chen, Shenxun Shi, Wei Chen, Hongyan Zhang, Ronghua Tang, Zhibing Wu, Yan Li, Jun Wu, Li Zong, Lianying Ji, Ping Feng, Jing Li

**Affiliations:** ^1^Mental Health Center, West China Hospital, Sichuan University, Chengdu, China; ^2^National Medical Products Administration Key Laboratory for Clinical Research and Evaluation of Innovative Drugs, Clinical Trial Center, West China Hospital, Sichuan University, Sichuan, China; ^3^Department of Psychiatry, Huashan Hospital, Fudan University, Shanghai, China; ^4^Mental Health, Sir Run Run Shaw Hospital, Zhejiang University School of Medicine, Hangzhou, China; ^5^Drug Clinical Trial Institution, Peking University Sixth Hospital, Beijing, China; ^6^Department of Neurology, Tongji Hospital, Tongji Medical College, Huazhong University of Science and Technology, Wuhan, China; ^7^Department of Encephalopathy, The First Affiliated Hospital of Guangzhou University of Traditional Chinese Medicine, Guangzhou, China; ^8^Department of Sleep Psychology, Guangdong Provincial Hospital of Traditional Chinese Medicine, Guangzhou, China; ^9^Department of Neurology, Peking University Shenzhen Hospital, Shenzhen, China; ^10^R&D Center, Beijing Beilu Pharmaceutical Co., Ltd., Beijing, China

**Keywords:** generalized anxiety disorder, Hamilton Anxiety Scale, traditional Chinese medicine, Jiuwei Zhenxin granules, alprazolam, blood urea nitrogen, serum creatinine

## Abstract

**Background:**

Generalized anxiety disorder (GAD) is a chronic disorder characterized by excessive, pervasive, persistent worrying that is difficult to control. Jiuwei Zhenxin granules may be safer and more effective than non-benzodiazepine anti-anxiety drugs for treating GAD. This study aimed to assess the efficacy and safety of Jiuwei Zhenxin granules alone or in combination with the benzodiazepine alprazolam.

**Materials and methods:**

A total of 710 patients were recruited from outpatient clinics and were randomly divided into two groups to receive Jiuwei Zhenxin granules (single drug group) or Jiuwei Zhenxin granules and alprazolam (combination group). The primary outcome was the response rate, which was defined as a ≥ 50% reduction from the baseline total score on the Hamilton Anxiety Scale (HAMA). Secondary outcome measures included mean changes in HAMA total score, psychological and somatic factors, Hamilton Depression Rating Scale total score, and SF-36 health survey score.

**Results:**

At 4 weeks after treatment, the single and combination treatment groups showed significant improvement in the HAMA total score and they did not differ significantly in response rate (77.58 vs. 79.17%) or rate of adverse drug reactions (16.22 vs. 16.07%).

**Conclusion:**

Jiuwei Zhenxin granules are an effective, safe, and well-tolerated treatment against GAD. Combining them with alprazolam may not significantly improve efficacy.

**Clinical trial registration:**

[www.ClinicalTrials.gov], identifier [CHICTR1800020095].

## Introduction

Generalized anxiety disorder (GAD) is a chronic disorder associated with pervasive and excessive worry that is difficult to control (1). GAD is often accompanied by non-specific physical and psychological symptoms, and the lifetime risk of GAD is about 6% in the general population (2–4). Several drug classes have been evaluated for their therapeutic efficacy in GAD, including benzodiazepines, azapirones, tricyclic antidepressants, selective serotonin reuptake inhibitors, and serotonin-norepinephrine inhibitors (3, 5, 6). Response to treatment is generally defined as a ≥50% reduction from the baseline total score on the Hamilton Anxiety Rating Scale (HAMA) (7, 8), based on which the clinical response rates range between 30 and 68% among GAD patients (6, 9, 10). In addition, several drugs currently used may increase the risk of adverse effects such as drug dependence, withdrawal syndrome, somnolence, gastrointestinal symptoms, and sexual dysfunction (11, 12). These considerations highlight the need for safer, more effective therapeutic approaches (6).

Jiuwei Zhenxin granules are a traditional Chinese remedy consisting of nine kinds of Chinese herbal medicines: *Panax ginseng C.A. Mey, Ziziphus jujuba Mil. var spinosa (Bunge) Hu ex H.F. Chou, Schisandra chinensis (Turcz.) Baill, Polygala tenuifolia Willd, Asparagus cochinchinensis (Lour.) Merr, Corydalis yanhusuo W.T. Wang, Paria cocos (Schw.) Wolf, Rehmannia glutinosa Libosch*, and *Cinnamomum cassia Presl* (13). They also contain various active ingredients, such as ginsenosides, *Rehmannia*-related polysaccharides, jujube seed alcohol, Poria sugar, and deoxyschizandrin, which have demonstrated anti-depressant, anti-anxiety, and neuroprotective properties in animal studies (14–16). Jiuwei Zhenxin granules were approved for the treatment of GAD by the Chinese National Medical Products Administration in 2008 (17). A relatively small phase II clinical trial in GAD patients showed that Jiuwei Zhenxin granules have greater therapeutic efficacy and fewer side effects than buspirone, a non-benzodiazepine anxiolytic drug (18). A later phase III trial showed that Jiuwei Zhenxin granules are similar in efficacy and safety to azapirones (18, 19). To the best of our knowledge, there are rare studies comparing the clinical efficacy and safety of this medication with benzodiazepine-based anti-anxiety drugs.

Therefore, in the present study, we performed a multicenter, randomized, parallel-group, controlled trial to evaluate the efficacy and safety of Jiuwei Zhenxin granules alone or in combination with the most common benzodiazepine, alprazolam, in patients with GAD.

## Materials and methods

### Inclusion and exclusion

A total of 710 patients were recruited from 12 hospitals across China. Patients were considered eligible for the study if they (1) were between 18 and 70 years old, (2) had been diagnosed with GAD based on the International Classification of Diseases (10th Revision) (20), and (3) had a baseline total HAMA score ≥14 and anxiety subscore ≥2. Patients were excluded if they had any of the following: other mental disorders associated with anxiety disorders, such as depression, terror-induced anxiety, panic disorder, obsessive-compulsive disorder, schizophrenia, or bipolar disorder; a total score ≥17 on the Hamilton Depression Rating Scale (HAMD); significant functional impairment of the heart, kidneys, or liver; or pregnancy or breastfeeding. There was no restriction on whether the subjects were outpatients or inpatients, or whether they were first treated.

### Sample size and randomization

Considering a statistical power of 80% and a significance level of 5%, we estimated a minimal sample size of 350 subjects per group, assuming a 10% difference in response rate and a 10–20% dropout rate. Patients were randomly allocated (1:1) to either a single drug group that received Jiuwei Zhenxin granules (6 g in the morning, 6 g at noon, and 6 or 12 g at night) for 4 weeks, or to a combination group that received Jiuwei Zhenxin granules (6 g in the morning, 6 g at noon, and 6 or 12 g at night) for 4 weeks, as well as alprazolam (0.4–0.8 mg bid or tid) for the first 2 weeks. Randomization was performed using the Proc Plan Procedure in SAS 9.2 (SAS Institute, Cary, NC, USA).

### Outcomes and measurements

The primary outcome of the study was the response rate, which was defined as a ≥50% reduction from the baseline HAMA total score at 4 weeks post-treatment. HAMA is used to assess anxiety symptoms and consists of 14 items scored on a five-point scale, ranging from 0 (absent) to 4 (severe) (21). Higher HAMA total scores indicate greater psychological distress and anxiety.

Secondary outcomes included mean changes in HAMA total score, psychological and somatic factors, HAMD total score, and SF-36 health survey score from baseline to endpoint (22). HAMA examinations were performed at baseline and at 2 and 4 weeks post-treatment. SF-36 health surveys were conducted at baseline and at 4 weeks post-treatment.

Adverse events in both groups were recorded, and their association with Jiuwei Zhenxin granules and alprazolam was classified as *related, probably related, possibly related, possibly unrelated*, or *unrelated*. *Related, probably related*, and *possibly related* events were considered adverse drug reactions.

Medical history, demographic characteristics, and physical examination results were recorded for all patients at baseline. Follow-up was conducted at 2 and 4 weeks after treatment. At baseline and at 4 weeks after treatment, all patients underwent blood, urine, and stool routine tests, and the levels of serum alanine aminotransferase, blood urea nitrogen, and serum creatinine were determined.

### Statistical analyses

Statistical analyses were performed with the SAS 9.2 software package based on a modified intention-to-treat approach (23). Data were expressed as mean ± standard deviation (SD) for continuous variables and as total number (% frequency) for categorical variables. Continuous variables were checked for the normality of distribution by a Kolmogorov-Smirnov test. If the normality test indicated normal distribution of the data, then a parametric test was used, otherwise, a non-parametric test was used. Paired *t*-test was used for the comparison of pre- and post-treatment within a group, and two-sample *t*-test was applied for comparison between two treatment groups in parameter analysis. Wilcoxon signed-rank test for the comparison of pre- and post-treatment within a group and Wilcoxon rank sum test for comparison between two treatment groups in non-parametric test. And differences in categorical variables were assessed using chi-squared or Fisher’s exact tests. Differences associated with *P* < 0.05 were considered statistically significant.

### Ethics and registration

The study was conducted according to the guidelines of the Declaration of Helsinki and Good Clinical Practice, and the protocol was approved by the local Ethics Committee [(2008 Clinical Trial (Post Marketing) Review (No.13)]. The trial was registered in the Chinese Clinical Trial Registry (registration no. CHICTR1800020095). Written informed consent was obtained from all patients before enrollment. The personnel who made disease diagnosis and performed scale assessments were qualified doctors and they had been uniformly trained at the start of the study.

## Results

**FIGURE 1:**
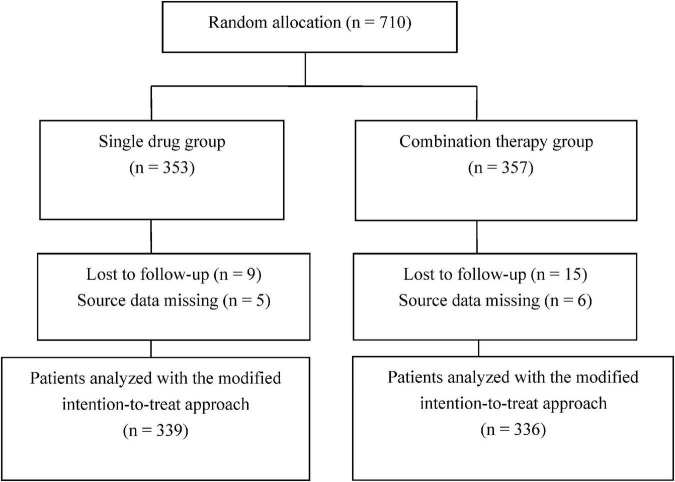
Flowchart of patient enrollment.

A total of 710 patients were enrolled in the present study, of whom 353 were assigned to the single drug group and 357 to the combination therapy group. Nine patients from the single drug group and 15 from the combination group were lost during follow-up, while necessary data were missing for 11 patients (Figure 1).

Patients from the two treatment groups were non-significantly different in age, gender, and body weight. Analysis of the clinicodemographic characteristics of the included patients revealed a statistically significant, but clinically unimportant, difference in blood lymphocyte count between the two groups (Table 1). The groups did not show any other significant differences in baseline clinicodemographic features or secondary outcomes (Table 2).

**TABLE 1:** Baseline characteristics of patients with generalized anxiety disorder treated with Jiuwei Zhenxin granules alone (single drug group) or in combination with alprazolam (combination group).

Characteristic*	Single drug group (*n* = 339)	Combination group (*n* = 336)	Statistics^a,b,c^	*P*-value
Age (year)	41.02 ± 12.98	41.76 ± 12.44	0.87^a^	0.382
Male	132 (38.94)	141 (41.96)	0.64^b^	0.726
Body weight (kg)	58.81 ± 9.77	59.20 ± 9.70	0.30^a^	0.767
Body temperature (°C)	36.39 ± 0.29	36.41 ± 0.30	1.00^a^	0.319
Pulse rate (beat/min)	75.20 ± 9.24	75.70 ± 9.68	0.58^a^	0.559
Systolic blood pressure (mmHg)	116.7 ± 12.30	117.0 ± 12.04	0.01^a^	0.991
Diastolic blood pressure (mmHg)	74.49 ± 8.30	75.48 ± 7.26	1.64^a^	0.101
Hemoglobin (g/L)	137.80 ± 14.60	138.65 ± 15.34	0.90^c^	0.369
Red blood cell count (10^12^/L)	4.64 ± 0.50	4.62 ± 0.51	−0.73^c^	0.465
White blood cell count (10^9^/L)	6.28 ± 1.69	6.28 ± 1.78	0.06^c^	0.952
Blood neutrophil count (%)	58.04 ± 8.62	58.84 ± 9.72	1.11^c^	0.267
Blood lymphocyte count (%)	33.27 ± 8.02	31.96 ± 8.75	2.01^a^	0.044
ALT (IU/L)	20.60 ± 13.05	21.67 ± 15.25	0.82^c^	0.410
BUN (mmol/L)	4.83 ± 1.37	4.85 ± 1.24	0.57^c^	0.571
CR (μmol/L)	74.48 ± 16.70	75.43 ± 17.12	0.56^c^	0.575

*Values are shown as *n* (%) or mean ± SD, unless otherwise indicated.

^a^Wilcoxon rank-sum test.

^b^Chi-squared test.

^c^*t*-test.

ALT, alanine aminotransferase; BUN, blood urea nitrogen; CR, serum creatinine.

**TABLE 2:** Comparison of baseline psychological and somatic factors between patients with generalized anxiety disorder treated with Jiuwei Zhenxin granules alone (single drug group) or in combination with alprazolam (combination group).

Measures	Single drug group* (*n* = 339)	Combination group* (*n* = 336)	Statistics^a,b^	*P*-value
HAMA total score	22.76 ± 5.85	22.94 ± 5.63	0.52^a^	0.600
HAMA psychic factor score	13.16 ± 3.59	13.17 ± 3.31	0.09^a^	0.930
HAMA somatic factor score	9.60 ± 3.39	9.77 ± 3.32	0.89^a^	0.375
HAMA items				
Anxious mood	2.76 ± 0.67	2.74 ± 0.68	−0.50^a^	0.616
Tension	2.25 ± 0.84	2.23 ± 0.80	−0.19^a^	0.848
Fears	1.09 ± 0.99	1.06 ± 0.95	−0.35^a^	0.729
Insomnia	2.57 ± 1.03	2.64 ± 0.93	0.39^a^	0.699
Cognitive	1.90 ± 1.05	1.91 ± 0.95	0.20^a^	0.843
Depressed mood	1.22 ± 0.81	1.18 ± 0.74	−0.33^a^	0.741
Somatic muscular	1.28 ± 0.97	1.28 ± 0.91	0.12^a^	0.904
Somatic sensory	1.28 ± 0.92	1.36 ± 0.90	1.32^a^	0.186
Cardiovascular	1.77 ± 0.84	1.83 ± 0.83	0.73^a^	0.465
Respiratory	1.40 ± 0.92	1.43 ± 0.86	0.41^a^	0.685
Gastrointestinal	1.35 ± 0.93	1.37 ± 0.87	0.64^a^	0.519
Genitourinary	0.88 ± 0.85	0.95 ± 0.87	0.91^a^	0.361
Autonomic	1.64 ± 0.89	1.55 ± 0.87	−1.37^a^	0.172
Behavior	1.36 ± 0.81	1.41 ± 0.84	0.68^a^	0.498
HAMD total score	11.09 ± 2.75	10.98 ± 2.94	−0.47^a^	0.638
SF-36 health survey	98.43 ± 13.54	99.32 ± 12.99	−0.88^b^	0.381

*Values are shown as mean ± SD.

^a^Wilcoxon rank-sum test.

^b^*t*-test.

HAMA, Hamilton Anxiety Scale; HAMD, Hamilton Depression Rating Scale.

**FIGURE 2:**
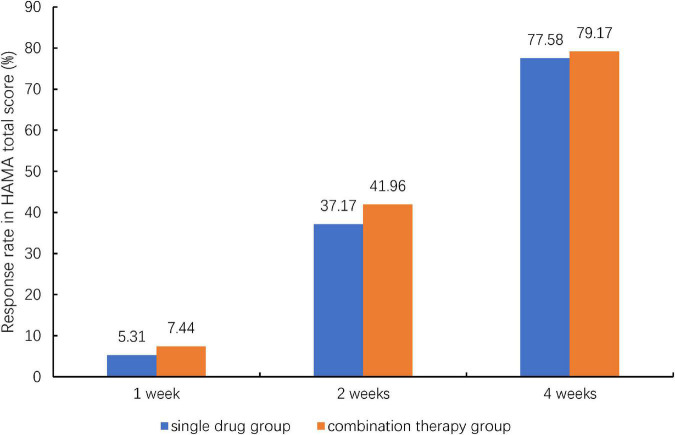
Therapeutic response rate of patients with generalized anxiety disorder to Jiuwei Zhenxin granules alone (single drug group) or in combination with alprazolam (combination therapy group) at 4 weeks post-treatment. HAMA, Hamilton Anxiety Scale; TCM, traditional Chinese medicine. Response was defined as a 50% reduction from the baseline HAMA total score at 4 weeks post-treatment. And Chi-squared test was used for the comparison between the two treatment groups.

Comparison of the HAMA total score at 4 weeks post-treatment indicated a better response in the combination group (77.58%) than in single drug group (79.17%), but the differences did not achieve statistical significance (*P* = 0.6169, Figure 2). The HAMA was improved continuously during the 4 weeks. Table 3 showed the comparison of HAMA and SF-36 health survey scores at baseline and at 4 weeks post-treatment. The mean change (±SD) for HAMA total score improved 13.09 (±6.52) for single drug group and 13.25 (±5.97) for combination group after 4 weeks treatment, the mean change for HAMA psychic factor score improved 7.17 (±3.77) for single drug group and 7.25 (±3.42) for combination group, and the mean change for HAMA somatic factor score improved 5.92 (±3.46) for single drug group and 6.00 (±3.21) for combination group. The improvements were statistically significant between baseline and post-treatment within each group. However, these changes were not statistically significant between the two groups (Table 3). Similar results were obtained for the SF-36 total score and its eight subscales.

**TABLE 3:** Comparison of HAMA and SF-36 health survey scores at baseline and at 4 weeks post-treatment between patients with generalized anxiety disorder treated with Jiuwei Zhenxin granules alone (single drug group) or in combination with alprazolam (combination group).

Measure	Single drug group (*n* = 339)			Combination group (*n* = 336)		
		
	Baseline	Week 4	Mean change	Baseline	Week 4	Mean change
HAMA total score^a^	22.76 ± 5.85	9.67 ± 5.11	13.09 ± 6.52^b^*	22.94 ± 5.63	9.69 ± 5.34	13.25 ± 5.97^b^*
HAMA psychic factor score^a^	13.16 ± 3.59	5.99 ± 3.06	7.17 ± 3.77^b^*	13.17 ± 3.31	5.92 ± 3.04	7.25 ± 3.42^b^*
HAMA somatic factor score^a^	9.60 ± 3.39	3.68 ± 2.75	5.92 ± 3.46^b^*	9.77 ± 3.32	3.77 ± 2.91	6.00 ± 3.21^b^*
SF-36 total score	98.43 ± 13.54	112.0 ± 12.26	13.55 ± 12.48	99.32 ± 12.99	111.7 ± 11.85	12.37 ± 11.97
Physical functioning^a^	27.25 ± 3.00	28.60 ± 1.80	1.35 ± 2.22	26.91 ± 3.39	28.35 ± 2.08	1.44 ± 2.45
Role physical^a^	5.51 ± 1.59	6.68 ± 1.48	1.17 ± 1.48	5.67 ± 1.57	6.69 ± 1.45	1.02 ± 1.55
Bodily pain^a^	8.91 ± 1.71	9.85 ± 1.20	0.94 ± 1.33	9.01 ± 1.64	9.73 ± 1.27	0.73 ± 1.14
General health^a^*	12.04 ± 3.36	16.16 ± 2.41	4.12 ± 3.10	12.49 ± 3.02	16.07 ± 2.26	3.57 ± 2.73
Vitality^a^	12.97 ± 3.47	15.73 ± 3.31	2.76 ± 3.28	13.44 ± 3.36	15.90 ± 3.28	2.45 ± 2.81
Social functioning^a^	7.68 ± 2.07	8.75 ± 1.61	1.07 ± 1.40	7.73 ± 1.77	8.70 ± 1.43	0.97 ± 1.34
Role emotional^a^	3.83 ± 1.10	4.85 ± 1.15	1.02 ± 1.18	3.83 ± 1.02	4.87 ± 1.12	1.03 ± 1.22
Mental health^a^	16.50 ± 4.07	19.99 ± 2.74	3.49 ± 3.71	16.60 ± 3.74	19.96 ± 2.62	3.36 ± 3.75

Values are shown as mean ± SD.

*P < 0.05.

^a^Wilcoxon rank-sum test for comparison between two treatment groups.

^b^Wilcoxon signed-rank test for the comparison of pre- and post-treatment within a group.

HAMA, Hamilton Anxiety Scale.

The rates of adverse events and adverse drug reactions were also similar between the two groups. The adverse event for single drug group was 16.22%, while for combination group was 16.07%. The adverse drug reaction for single drug group was 8.26 and 11.01% for combination group (Table 4). The most common adverse drug reactions were dry mouse (3.24%) and abdominal discomfort (1.77%) in the single drug group; or abdominal discomfort (2.08%), constipation (1.19%), diarrhea (1.49%), and dizziness (1.79%) in the combination group (Table 5).

**TABLE 4:** Rates of adverse events and adverse drug reactions in patients with generalized anxiety disorder treated with Jiuwei Zhenxin granules alone (single drug group) or in combination with alprazolam (combination group).

	Single drug group (*n* = 339)	Combination group (*n* = 336)
**Adverse event**		
Yes	55 (16.22)	54 (16.07)
No	284 (83.78)	282 (83.93)
**Adverse drug reaction**		
Yes	28 (8.26)	37 (11.01)
No	311 (91.74)	299 (88.99)

Values are *n* (%).

**TABLE 5:** Adverse events and adverse drug reactions occurring in >1% of patients with generalized anxiety disorder who were treated with Jiuwei Zhenxin granules alone (single drug group) or in combination with alprazolam (combination group).

	Single drug group (*n* = 339)	Combination group (*n* = 336)
**Adverse event**		
Dry mouth	11 (3.24)	1 (0.30)
Abdominal discomfort	6 (1.77)	7 (2.08)
Constipation	1 (0.29)	4 (1.19)
Diarrhea	0 (0)	5 (1.49)
Dizziness	5 (1.47)	6 (1.79)
Headache	4 (1.18)	2 (0.60)
Urine red blood cell positive	5 (1.37)	2 (0.60)
Urine white blood cell positive	3 (0.88)	7 (2.08)
Total	55 (16.22)	54 (16.07)
**Adverse drug reaction**		
Dry mouth	9 (2.65)	1 (0.30)
Abdominal discomfort	4 (1.18)	5 (1.49)
Constipation	1 (0.29)	4 (1.19)
Diarrhea	0 (0)	4 (1.19)
Dizziness	3 (0.88)	4 (1.19)
Total	28 (8.26)	37 (11.01)

Values are *n* (%).

## Discussion

Generalized anxiety disorder is a prevalent disorder associated with significant impairments in social, emotional, and physical functioning, and it has received increasing attention in recent years. Jiuwei Zhenxin granules have been approved for the treatment of patients with GAD, but their safety and therapeutic efficacy have been compared mainly to non-benzodiazepine drugs (24, 25). In the present multicenter, randomized controlled study, we evaluated for the first time the safety and efficacy of Jiuwei Zhenxin granules in the presence or absence of the benzodiazepine anxiolytic drug alprazolam. Our results showed that as monotherapy or in combination therapy, Jiuwei Zhenxin granules can effectively relieve GAD without severe adverse events.

Benzodiazepines are considered the primary pharmacological treatment for GAD (26), with alprazolam being the most frequently prescribed agent (27). Jiuwei Zhenxin granules have also been identified as a promising treatment for GAD (24), and their efficacy has been confirmed in phase II and III trials (18, 19). Indeed, Jiuwei Zhenxin granules can significantly reduce HAMA total score in GAD patients (10, 28). Consistent with these results, we found that this traditional medicine, either alone or combined with alprazolam, can greatly improve the HAMA total score. In contrast to our findings, previous trials showed that combining the granules with buspirone (29) or escitalopram (29, 30) was more effective at reducing the HAMA score than the corresponding monotherapies. This discrepancy may be due to differences in the drugs’ mechanism of action, and the fact that we excluded patients with HAMD total score ≥17, which was lower than the threshold used in those previous studies.

The incidence of adverse effects associated with Jiuwei Zhenxin granules was reported to be lower, albeit not significantly so, than the incidence with co-administration of buspirone and granules (30%) or with buspirone alone (25%) (28). Here, the adverse drug reaction rate was 8.26% for the single drug group and 11.01% for the combination group, which was lower than the values previously reported. Further research could explore the safety of benzodiazepine and non-benzodiazepine when combined with the granules.

Our study had certain limitations. One is that we excluded patients with HAMD score ≥17, so whether our findings can be extrapolated to a broader range of GAD patients needs to be confirmed. Another limitation is that the rate of therapeutic response differed by less than 10% between the two groups, suggesting that our results need to be confirmed in a larger sample. In addition, the course of disease before the enrollment was not recorded.

## Conclusion

The present study suggests that Jiuwei Zhenxin granules is safe and effective in treating GAD. Nevertheless, our results should be validated in future studies with larger samples and higher HAMD thresholds.

## Data availability statement

The original contributions presented in this study are included in the article/supplementary material, further inquiries can be directed to the corresponding authors.

## Ethics statement

The studies involving human participants were reviewed and approved by the Independent Ethics Committee of West China Hospital. The patients/participants provided their written informed consent to participate in this study.

## Author contributions

PF and JL contributed to the conceptualization and design of the study. XW, SS, HZ, WC, RT, ZW, YL, and JW conducted the trial in a separate center. PF analyzed the data. XW and PC wrote the manuscript. LZ and LJ reviewed and revised the manuscript. All authors critically interpreted the results and approved the final version of the manuscript.

## Abbreviations

GAD: generalized anxiety disorder
HAMA: Hamilton Anxiety Scale
HAMD: Hamilton Depression Rating Scale
SD: standard deviation
TCM: traditional Chinese medicine.
